# Daily life without cranial bone protection while awaiting cranioplasty: a qualitative study

**DOI:** 10.1007/s00701-024-06217-5

**Published:** 2024-08-09

**Authors:** Henrietta Gustavsson, Eva Jangland, Lena Nyholm

**Affiliations:** 1 Department of Medical Sciences, Neurosurgery, Uppsala University, Uppsala, Sweden; 2 Department of Surgical Sciences, Nursing Research, Uppsala University, Uppsala, Sweden; 3 Uppsala University Hospital, Entrance 85, NIVA, 751 85 Uppsala, Sweden

**Keywords:** Decompressive craniectomy, Craniotomy, Neurosurgery, Critical care, Qualitative research

## Abstract

**Purpose:**

Decompressive craniectomy is occasionally performed as a life-saving neurosurgical intervention in patients with acute severe brain injury to reduce refractory intracranial hypertension. Subsequently, cranioplasty (CP) is performed to repair the skull defect. In the meantime, patients are living without cranial bone protection, and little is known about their daily life. This study accordingly explored daily life among patients living without cranial bone protection after decompressive craniectomy while awaiting CP.

**Methods:**

A multiple-case study examined six purposively sampled patients, patients’ family members, and healthcare staff. The participants were interviewed and the data were analyzed using qualitative content analysis.

**Results:**

The cross-case analysis identified five categories: “Adapting to new ways of living,” “Constant awareness of the absence of cranial bone protection,” “Managing daily life requires available staff with adequate qualifications,” “Impact of daily life depends on the degree of recovery,” and “Daily life stuck in limbo while awaiting cranioplasty.” The patients living without cranial bone protection coped with daily life by developing new habits and routines, but the absence of cranial bone protection also entailed inconveniences and limitations, particularly among the patients with greater independence in their everyday living. Time spent awaiting CP was experienced as being in limbo, and uncertainty regarding planning was perceived as frustrating.

**Conclusion:**

The results indicate a vulnerable group of patients with brain damage and communication impairments struggling to find new routines during a waiting period experienced as being in limbo. Making this period safe and reducing some problems in daily life for those living without cranial bone protection calls for a person-centered approach to care involving providing contact information for the correct healthcare institution and individually planned scheduling for CP.

**Supplementary Information:**

The online version contains supplementary material available at 10.1007/s00701-024-06217-5.

## Introduction

Decompressive craniectomy (DC) is a life-saving neurosurgical intervention when a large portion of the skull bone has been removed to prevent secondary injuries such as ischemic brain and brain herniation in patients with acute severe brain damage. DC reduces refractory intracranial hypertension, as a last-tier treatment, by creating space and restoring adequate oxygenation [21]. Traumatic brain injury, malignant stroke, and intracerebral hemorrhage are the most common underlying diagnoses requiring DC [15]. Following DC, cranioplasty (CP), a surgical procedure to repair the skull defect, is required [15].

Although DC may improve survival, the evidence for outcome is inconsistent [5, 6, 12, 14, 21]. Furthermore, the subsequent CP is associated with additional risk of certain complications such as infections, intracranial hemorrhage, and extra-axial fluid collections [8, 18, 26]. Sunken flap syndrome is another complication of DC, in which the atmospheric pressure is thought to affect the skin flap, leading to neurological deficits [8, 21]. Studies suggest that the patient’s neurological status may improve following CP when vascular and cerebrospinal circulation are normalized [8, 21].

The optimal timing of the procedure, however, remains controversial and is a balancing act between neurological recovery and the risk of complications [17]. Yet another factor affecting timing is the strained situation that public healthcare is facing, and during the time between DC and CP many patients are discharged from hospital without cranial bone protection.

DC and the subsequent CP have been studied from perspectives of neurological outcome and complication rates from a surgical point of view, but there is limited knowledge of how daily life in the absence of cranial bone protection is experienced by the patients [31, 33]. To our knowledge, no qualitative studies have explored the patient experience and perspective. To evaluate all aspects of CP following DC, it is crucial to include the patient perspective.

## Aim

The aim of this study was to explore daily life among patients living without cranial bone protection after DC, while awaiting the subsequent cranioplasty.

## Methods

### Design

This qualitative study was performed using a multiple-case design, enabling us to examine a phenomenon from different points of view and using different information sources. Interviewing individuals from different contexts in a multiple-case study enables analyses both within and across cases. Examining similarities and differences among cases will deepen our understanding of the daily-life situation of patients in the absence of cranial bone protection [1, 32]. This study follows the Consolidated Criteria for Reporting Qualitative Research (COREQ) for interviews and focus groups [28].

### Participants

The cases were built around seven patient cases; the interviewees were six patients, six of their family members, and three primary nurses (see Table 1). One contacted patient decided not to participate in the study, but no reason for this was asked for. The patients were adults who had undergone DC as a life-saving procedure due to acute severe brain injury and were waiting for reconstructive CP. Table 2 shows the characteristics of the patients.

**Table 1:** Overview of cases

Case	Interviewed
1	Family member and primary nurse
2	Patient and family member
3	Patient, family member, and primary nurse
4	Patient and family member
5	Patient and family member
6	Patient and family member
7	Patient and primary nurse

**Table 2:** Characteristics of the patients

Age in years, range (median / range)	52 / 33–64
Sex (women / men)	4 / 3
*Living situation:*	
Living alone with homecare service	2
Cohabitation with family member or friends	3
Inpatient rehabilitation	1
Nursing home	1
*Diagnoses requiring DC:*	
Intracerebral hemorrhage	3
Traumatic brain injury	2
Malignant stroke	1
Subarachnoid hemorrhage	1
Location of DC (left / right)	4 / 3
Aphasia or dysphasia	4
Vegetative	1
GCS-M^a^ score at admission to NICU (median / range)	5 / 1–6
GCS-M^a^ score before DC* (median / range)	5 / 2–6
GOS-E^b^ at time of interview (median / range)	4 / 2–6
Time from initial DC to interview, months (median / range)	15 / 2–24

^a^ Glasgow Coma Scale—Motor Score [27]

^b^ Glasgow Outcome Scale-Extended [30]

^*^Last GCS followup before DC

Since the patients had brain damage due to their initial injury, some of them subsequently had difficulties speaking or remembering things. That is why we also chose to interview family members and healthcare staff regarding the patients’ daily life. The included family members were in regular contact with the patients, so they could reflect on the patients’ daily life.

DC affects the patients’ appearance, causing asymmetric head shape due to scalp retraction and perhaps conspicuous scarring. The patients were advised to wear a helmet to protect the unshielded brain when moving, which was obviously salient. The helmet used is usually made of plastic and looks like a bulky sports helmet.

All included patients were treated at the neurointensive care unit (NICU) at a university hospital in Sweden. The patients underwent DC between 2021 and 2023 because of life-threatening high intracranial pressure (ICP) that could not be treated in any other way. At the time of the interviews, all patients had been discharged from hospital, except for one patient receiving inpatient rehabilitation. The patients had been waiting between two and 24 months for DC at the time of the interviews. Two patients had undergone CP but, due to infection, had the new bone flap removed one week and two months post surgery, respectively. Before the initial injury, all patients were working and managed their daily life independently.

The patients were identified by the first author (HG) and LN from the neurosurgical department’s waiting list of patients eligible for reconstructive CP, and selected purposefully to obtain variation in diagnosis, neurological recovery, age, gender, and time passed since initial surgery.

### Settings

Most patients with acute severe brain injury at the current NICU are referred from their local hospitals where they received the initial emergency treatment. Most patients are sedated, mechanically ventilated, and have multimodal monitoring including ICP registration. If needed, DC is performed as a life-saving procedure. When specialized medical care is no longer needed, patients are transferred back to their local hospitals for further treatment and rehabilitation while awaiting reconstructive CP. Patients are eventually discharged from hospital during the waiting period.

### Data collection

A letter containing brief information about the study was sent to the patients, informing them that they were about to receive a telephone call regarding participation. Since the researcher who intended to conduct the interviews was employed at the NICU when the patients were being treated there, a research team member (LN) having no previous professional relationship with the patients first called each patient or a family member to ask whether it would be all right to be contacted by HG. If agreement was obtained, HG contacted that same person to plan who would be suitable to be included in that particular case, and to schedule the time and setting of the interview. Participants were recruited continuously during the interview process. After interviewing six cases no new categories were found. We included one more case, and as nothing new emerged data saturation was considered to have been reached [2].

Data were collected through interviews. Fifteen interviews were conducted, six with patients, six with family members, and three with primary nurses (see Table 1). The first author (HG) conducted the interviews from October 2022 to June 2021. The interviews were semi-structured with open-ended questions intended to elicit descriptions of daily life without cranial bone protection in everyday life situations that could conceivably be affected. A documentation model [7] was used to identify areas of questioning for the interview guide. The model used is a checklist for nursing documentation and covers all aspects of daily life. To test the interview guide, a pilot interview was conducted beforehand, and no substantial changes were deemed necessary (see Supplementary material for the interview guide), although minor changes were made to the questions during the interview process.

All interviews were individual except for two in which the patients wanted a family member to attend for support. The patients were interviewed in their homes or at a rehabilitation unit, except for two who were interviewed by telephone. The family members were interviewed in their homes (2), by telephone (3), or at the interviewer’s workplace (1). Staff were interviewed at the patients’ locations. All interviews were audio-recorded and transcribed verbatim by the first author (HG); however, the transcripts were not returned to the participants, who had no opportunity to provide feedback on the results. The interviews lasted 5–60 min. The shorter interviews were with staff who had little to add about the subject and with a patient with severe speech impairment, who could only communicate through gestures and single words. The longer interviews were either rich in data or fragmented and addressing matters other than those asked about.

Due to the communication impairment of the participating patients, field notes were taken describing gestures and behaviors to clarify their messages as well as occasional use of wrong terms due to aphasia. This made the non-verbal data understandable for the other authors. In addition, field notes were made regarding, for example, the environment, patient position during the interview, use of aids, disruptive elements during interviews (e.g., sounds of alarms or television, presence of other persons), voice pitch, and pauses. At the end of the interviews, the interviewer summarized what had been said so that the participants could agree or disagree and add more content if they wished.

### Analysis

Data were analyzed using qualitative content analysis [9, 10], which is a qualitative method suitable for analyzing text from interviews, narratives, and observations intended to describe peoples’ experiences and perceptions of a certain phenomenon. Qualitative content analysis explores variations in the phenomenon of interest, revealing similarities and differences in the data, and was therefore a suitable method for this study. All three authors participated in the analysis process (see details in Table 3).

**Table 3:** Process of analysis and pre-understanding

Step in analysis	Description of actions
Familiarizing with data	The recorded interviews were listened to and the transcripts (and field notes) were read carefully by the authors to get a sense of the whole
Coding of data	The text was then condensed and coded independently by HG and LN, without using software. The authors compared their coding schemes, which were found to be mainly coherent. After minor revisions, the authors agreed on what codes addressed the aim
Seeking categories and excluding data not addressing the aim	Codes with similar contents were sorted into categories, which were further interpreted and abstracted
Reviewing and defining categories	The analysis process entailed continuous discussion among all authors and making comparisons with the original text, leading to the adjustment of the categories until the researchers agreed on the final result. Quotations illustrating the findings were chosen jointly by the authors
Pre-understanding and experience	As the researchers’ pre-understandings could have influenced both the interviews and the analysis process, it was necessary to be aware of them [9]. The authors’ pre-understandings were discussed continuously during the analysis. The first author (HG) is a female PhD student and a critical care nurse employed at the NICU where the patients were initially treated. She has several years’ experience of working with patients with acute brain injury but limited experience of the rehabilitation field and of performing interview studies and qualitative content analysis. The second (EJ) and last (LN) authors are female associate professors and senior lecturers in nursing and are well experienced in qualitative content analysis. The last author is also well experienced in working with this group of patients as a critical care nurse in an NICU. The second and third authors had no previous contact or professional relationship with the participants. Because the first researcher was employed at the NICU when the patients were treated in it, there is a small chance that they might have met briefly

## Ethics

The study followed the Code of Ethics of the World Medical Association (Declaration of Helsinki [34]) and was approved by Swedish Ethical Review Authority (2022–02696-01). Informed consent was obtained from the participants included in the study. In cases in which the patients were unable to give consent, assent were given by a family member.

## Results

The cross-case analysis identified five categories describing the daily life of the patients without cranial bone protection (see Table 4 for an overview). The participants within each case were mostly concordant in their answers, except from the degree of worrying reported. However, cross-cases the answers had a greater variation due to the patients being in different phases of their recovery. Depending on the grade of recovery and time passed since initial injury, the absence of cranial bone protection had varying impacts on the patients’ daily life. All patients suffered some sequelae of their initial brain injury, affecting their daily life by creating a changed life situation. All aspects within each category are presented below and illustrated by translated interview quotations supporting the findings, with comments from the field-notes presented in brackets. Some quotations have been edited for clarity to remove irrelevant phrases.

**Table 4:** Overview of results

Category	Content
Adapting to new ways of living	Wearing a helmet
	Sleeping position
	Pain and discomfort
	Need for an attendant
	Reduction in daily activities
	Appearance
Constant awareness of the absence of cranial bone protection	Risk of injury
	Family members worrying
Managing daily life requires available staff with sufficient qualifications	Constant presence of staff
	Availability of educated staff
Impact on daily life depends on degree of recovery	
Daily life ends up in limbo while awaiting cranioplasty	

### Adapting to new ways of living

Having no cranial bone protection caused patients to form new habits and strategies to cope with their new daily life, such as wearing a helmet, sleeping, and dealing with pain and discomfort, to overcome everyday challenges. According to the patients, minor inconveniences could be addressed without any great difficulty, although the absence of bone protection sometimes led to negatively charged situations, such as awareness of altered appearance or risk of injury. The patients and their family members were mostly satisfied with the acquired knowledge needed to cope with situations in daily life without cranial bone protection. However, they said that they could not fully remember the information given during the hospital stay.

***Wearing a helmet*** was described as having a great and significant impact on daily life*.* Although the helmet was not very comfortable, its purpose mostly justified wearing it. Some patients found it bothersome as it was perceived as heavy and hot and chafed the skin. It was also described as ugly, or embarrassing, bringing an additional challenge to daily life. Most patients intended to always wear the helmet, although they sometimes forgot it, especially as balance and bodily functions improved. Some participants also described seizing the chance to take it off at the earliest opportunity, while others said that they had completely stopped wearing it. One patient described a reason for taking it off:

My helmet is also quite heavy, so my neck gets tired and numb.

(Patient B)

Patients described how several adjustments had to be made to achieve the best helmet fit, although it remained a compromise because the shape of the head varied depending on position and the time elapsed since DC. The helmet was perceived to fit better and was of less concern after the initial swelling had gone down*.* Patients described the presence of the helmet as more noticeable than the absence of bone protection in everyday living. Patients and staff said that physical therapy worked out well but required strict helmet wearing or adjusted exercises to protect the area with no bone protection. However, extensively wearing the helmet when moving around became a habit, and the patients described developing new routines to always keep the helmet nearby and within easy reach.

And when I went to my daughter’s house, I wore it all the time … until I lay down on the sofa … . Then I put it away … . And then, when I got up I immediately put it on!

(Patient A)

A patient with dysphasia talked about routines when showering:

I’ll go in here and sit on this [pointing at a freestanding shower seat in the shower cabin]. Then I take this thing [the helmet] off and hang it here [points to a hook on the wall], without having to stand up. When I’m done I’ll just grab it again, and this actually works really well.

(Patient F)

Another adaption and challenge brought into daily life concerned sleeping. The patients talked about ***sleeping position*** and said that they needed to sleep only on their back or on the opposite side from the one with no cranial bone protection. Some patients said that they slept well, but that it would feel good to be able to shift position. The unpleasant feeling when touching the boneless area prevented some of the patients from accidentally turning towards the side with no cranial bone protection.

However, other patients complaining of poor sleep due to the absence of bone protection, or said that previous sleeping disorders worsened. One patient had been told to wear the helmet around the clock, which made it difficult for her to get proper sleep. Fear of accidentally turning towards the side with no bone protection led to not daring to shift positions at all, causing some patients to sleep only in a supine position, as described below:

Interviewer: But do you always sleep on your back? You don’t accidentally turn over in your sleep?

Patient: No, oh no. No, I lie still! … I’m afraid!

(Patient D)

This situation was unfamiliar to others, who described sleeping on their boneless side without worrying or feeling discomfort, and not recalling any information about restrictions or risks associated with this. Some patients and their family members said that an increased need for sleep due to fatigue, combined with difficulties resting or sleeping properly, led to additional weariness.

Accidentally touching the area with no cranial bone protection caused ***pain and discomfort*** for some patients. Painful chafing of the skin on the edges of the skull beneath the scalp was also described by the patients. Patients said that daily bathing and cleaning routines were challenging. In particular, washing their hair and getting a haircut were unpleasant, but mostly worked out well with a careful approach. Because of this, patients considered that hair washing was preferably performed by oneself without interference from others. However, some thought it was difficult to get their hair properly clean because of the unpleasantness. The helmet made the patients sweaty, and the patients said that they sometimes wanted to wash their hair more often, but the unpleasantness made them avoid it.

You become well aware of it the moment you touch it, because it hurts.

(Patient E)

Family members and staff also noticed that patients with impaired speech showed signs of discomfort and pain when the area was touched. A primary nurse described a showering situation with a patient who could not speak but clearly expressed himself in body language:

You know, the side of the head with no bone, you can say the most dangerous situation is when we help him to take a shower. You have to be really cautious about it … It is really sensitive. We notice … that it is so sensitive to him that we barely can touch it … We do it very carefully.

She further described his wide-eyed expression, apparently of alarm, and that they avoided rubbing the area:

I don’t know how it feels for him … but maybe he is troubled, scared, or in pain. We are really, really careful. We hardly dare to touch it.

(Primary nurse P)

She explained that she had noticed that the patient did not display this particular expression when washing the other side of the head, or the rest of his body.

Another troublesome aspect expressed by some patients was wearing glasses, which was considered somewhat painful as well as being hampered by swelling and the fact that the jaw muscle had moved due to the surgery or the presence of the helmet.

Balance problems subsequent to the brain damage entailed an increased risk of falling and injuring the unprotected brain. Patients mentioned using aids such as walkers to prevent falling, but also other strategies, such as putting a chair beside the bed or using a shower seat. Sometimes the risk of falling led to a ***need for an attendant*,** which was sometimes found rather limiting and to restrict spontaneity in activities. The absence of cranial bone protection led to a ***reduction in daily activities***. Both patients and family members pointed out that the absence of bone protection hindered daily activities, such as using the toilet without assistance, and performing leisure activities, such as traveling, ice-skating, or taking long walks unassisted:

There is no one who would dare let me near a horse as long as I do not have bone protection … which I long for, of course.

(Patient B)

The sunken scalp and wearing of a helmet entailed an ***appearance*** deviating from the norm. This was described as not a big issue by some patients, while others found it bothersome and avoided social gatherings or leisure activities, leading to social exclusion:

I’m actually … actually invited to a wedding in June, and I said to *X* “I’m not going if I don’t have anything here [pointing at the area without bone protection with only short and thin hair covering it]. I won’t do it!”

(Patient A)

Some patients saw appearance as irrelevant, not even having discussed the issue with their family or caring staff, while others said that they had become used to their appearance as time went by:

When I glance at myself in a [mirror] it actually looks like bloody hell. But when I get outside, I forget about the whole thing.

(Patient D)

Strategies such as trying to cover the boneless area by adjusting their hair or wearing a hat were described, and sometimes patients had received comments about their looks or noticed people gazing, but stated that it did not affect them:

And then when we sat down some people said “Oh my god, what does your head look like!”

(Patient A)

### Constant awareness of the absence of cranial bone protection

The absence of bone protection was described as something always on the patients’ minds, and the fact that the brain was relatively unprotected led to caution in daily life. This constant awareness also came from painful sensations when accidentally touching the area. The fact that the missing piece of bone was noticeably large contributed to this awareness. The rigorous wearing of the helmet was a result of this awareness, as was the choice not to perform leisure activities that incurred increased ***risk of injury***.

However, this awareness was seldom associated with worrying. Patients described having little fear of falling or hurting themselves, even though their balance was somewhat impaired, and several had even fallen on occasion. They trusted their bodily functions, and as the recovery proceeded, any possible concerns diminished. They also trusted the protective ability of the helmet.

The family members thought that the patients were anxious, but in fact it seemed as though it was mainly the ***family members worrying***. Their biggest fears were that the patients might fall, for instance, due to epileptic seizures, or that they would be lying injured for a long time before anyone noticed. The presence of another person standing by made the family members less worried. Since family members seemed somewhat more anxious about the patients’ situation than were the patients themselves, situations occurred in which family members demanded homecare service or constant attendant presence, whereas the patients themselves thought this was unnecessary, considering it somewhat of an overreaction that limited their daily activities:

You are well aware that there is nothing protecting it … so you just want someone to be there with him, or just coming over every day to check in on him … . Just to see that everything is okay.

(Family member L)

The family members mentioned that there was the possibility that the patients suffered from impaired memory as well as impaired insight into their situation, causing carelessness, leading to increased anxiety among family members. However, both patients and family members said that their anxiety decreased over time, when they noticed that daily life was working out well:

Family member: I sense that it’s mostly me, myself, limiting him. I’m like, you know … He was on his way up a ladder once, and I just ... “Dad, you are not allowed to do that!” That sort of thing. It’ s like … something might happen to me as well, but you are much more unprotected without the skull bone. So it often occurs that I tell him, “Dad, think about this once again.”

Interviewer: Do you think you worry more than he does?

Family member: Yes, I believe so.

(Family member L)

### Managing daily life requires available staff with sufficient qualifications

Varying grades of deficits following the brain injury entailed different living situations as well as varying demands for assistance in daily life, leading to discordant reports of the impact of the absence of cranial bone protection. For some patients, the absence of cranial bone protection alone justified the ***constant presence of staff***. Family members and staff described that other patients dependent on large-scale help efforts in everyday life ended up in situations in which the care-giving staff needed sufficient knowledge pertaining to the absence of cranial bone. This entailed rigorous staff scheduling to ensure the ***availability of educated staff***. Staff members said that they sometimes were obliged to obtain specific training to care for the patients who had undergone DC, but not all were willing to fulfill the requirements due to fear and unease. Also, family members said that homecare service staff sometimes felt insecure and were not always willing to care for patients with no cranial bone protection. Better information about daily care was sometimes wished for by the home care staff interviewed. Family members noticed that hair washing was not always properly performed and could see when care staff felt uneasy or insecure, leading them to distrust their ability and feel forced to assume some tasks themselves. However, well-educated staff who had received proper information felt secure and did not perceive any difficulties in assisting in daily care:

All the staff of course know that that he can never lie on the right side of his head … . Otherwise, you won’t get the delegation. … Everyone must know these things. Before the nurse gives you the delegation, all staff must be aware of these things about the patient.

(Primary nurse P)

### Impact on daily life depends on degree of recovery

In the new daily life routine, struggling with deficits and difficulties related to the acquired brain injury, the absence of cranial bone protection was not always the major concern and was sometimes described by the patients as just one problem among others. This was also confirmed in the interviews with family members and staff and seemed to be more prominent if the consequences of the brain injury had a major impact on independence and functioning in daily life.

Some patients said that the new situation had become familiar, in that the absence of bone protection was more disturbing at the beginning, before they got used to it. Conversely, other patients said that as recovery progressed with the improvement of functions, the absence of bone protection became more prominent as well as more limiting, preventing them from living the way they wished.

Patients with more severe deficits seemed to be less affected by the skull defect. In those cases, problems such as speech deficits, degraded motor skills implying dependence on assistance or aids, and fatigue were more significant in daily life, as described by patients, family members, and staff. It was difficult for some patients to separate deficits related to the brain injury from aspects concerning the absence of bone protection.

A patient with great dependence of assistance due to hemiparesis and his caregiver described mobilization in a wheelchair.

I can’t say that [the lack of bone protection] affects him very much … . Of all the things we do here, there are other things that are more difficult.

(Primary nurse O)

It’s other things that limit me [than the lack of bone protection] … . The fact that I get pain in my behind, that’s what affects how much [I can sit up].

(Patient E)

### Daily life ends up in limbo while awaiting cranioplasty

The patients considered the situation of having no cranial bone protection to be temporary, and they anticipated the upcoming surgery to restore the cranial structure so that they could go on with their lives. One patient described needing new glasses since his vision had changed, but said that no glasses fit properly when he was wearing the helmet, so he postponed the purchase. Uncertainty regarding the timing of surgery also made it difficult for the patients to plan their future. All patients had received information that they would have the surgery approximately three months after their injury, but several had been waiting for a longer time and had not been given any information as to why or how long they would have to wait. This change in perceived scheduling caused stress and anxiety, besides difficulties in planning daily life. Scheduled activities were inhibited or postponed, as the patients thought that the time of surgery would be announced shortly. Some patients described simply having to accept the uncertainty and put up with what the healthcare service told them to do:

But I hope [the surgery] will be before midsummer, so that I can be there then … in my camper.

(Patient A)

The waiting time was seen as too long and bothersome; at the same time, the patients expressed feelings of nervousness and worry about the surgery and anesthesia and avoided thinking about them. Complications such as a new stroke or infections were intimidating. Those already suffering from complications expressed great anxiety over the risk of complications happening again, especially if the emerging complications were caused by long waiting times. Concerns about a protracted, strenuous convalescence were also expressed. One patient expressed anxiety about the fact that another surgery would entail having one’s hair shaved again. Several had had their appointments for surgery considerably delayed, but they accepted the waiting and not being able to influence it. A long waiting time for potential medical reasons and priorities was more accepted than just the fact that there was a long waiting list:

It’s just the thing that [the bone] has to be put back … And you kind of want to have it done … Because, then it’s the recovery process. I guess it won’t be done in a trice.

(Family member K)

Not getting information about the surgery caused the patients and their families to try to contact the healthcare service, which was difficult to do. When they eventually succeeded, they perceived the reply as short or did not gain any actual information. The participants said that one would have to be lucky to talk to the right person to have any chance of affecting the waiting time. The nonexistent explanations and information were a source of irritation and frustration. Better communication about the waiting time and planning of surgery was desired and would simplify activity planning in daily life and reduce anxiety. Not being contacted regarding the upcoming surgery led to disappointment. When they were not scheduled for surgery, the patients described feeling that the healthcare service had turned its back on them. Questions about how the surgery was to be performed also came up during the interviews:

When we discussed it with the surgeon, he said that he hoped it would be done by summer… Well, this was last year.

(Family member I)

## Discussion

### Discussion of results

The patients living without cranial bone protection coped with daily life by developing new habits and routines, but on the other hand the absence of cranial bone protection also entailed inconvenience, such as the continuous need to wear a helmet. The impact of the absence of bone protection on daily life seemed to change over time, depending greatly on the patients’ other symptoms derived from the initial brain injury leading to discordant reports of experiences. The absence of bone protection was also described as just one of many drawbacks, especially in patients with a poor outcome. The waiting time for surgery was perceived as long and bothersome, particularly when the patients had not been provided with any information as to why or how long they would have to wait, together with difficulties contacting the healthcare system.

Wearing a helmet quickly became a habit for some patients in our study, although it was somewhat bothersome for others. Some patients therefore refused to wear it, and several patients said that they had fallen on occasion. There appears to be limited empirical research about head protection when living without cranial bone protection, or about how recommendations to wear helmets are followed. Obviously, there is a risk of brain damage if a patient without bone protection chooses not to wear a helmet, especially if falling. Only one case study was identified in this area [11] presenting a patient without cranial bone protection who fell to the ground and subsequently died from his injuries. Other studies in this area report that physical therapists felt more confident rehabilitating patients lacking bone protection if they wore helmets [3]. Pandit et al. [24] found that patients often accidentally lay on the side without bone protection. Our findings confirm that it is crucial that the helmet, first, should be well fitted. Second, we recommend that patients, family, and caring staff should be well educated and have ongoing access to information about the importance of wearing and how best to wear the helmet and protect the area without bone cover. However, that recommendation does not come without challenges, because this patient group has problems remembering verbal information.

The patients described in different ways being restricted in their daily lives by living without bone protection. This could apply to activities they wanted to but could not undertake and to their appearance causing them to refrain from social events. In a review regarding quality of life following CP, Mustafa et al. [22] demonstrated that CP may improve mental health due to improvement in cosmetic appearance, social functioning, and pain, but future validated condition-specific studies are required [22, 33]. In our study it seemed as though the severity of the symptoms associated with the brain injury affected how much concern the absence of bone protection entailed. Not having bone protection was perceived as restrictive among those who had greater independence in their everyday living. For these patients, spontaneity and the opportunity to perform activities could be further inhibited by the presence of an attendant or by family member worries. This was experienced as particularly frustrating when not knowing when surgery would be performed and not succeeding in contacting someone in healthcare who could provide that information.

There is no consensus in the literature about when CP should be performed [4, 13]. Nevertheless, based on the present results, person-centered care [20] could alleviate discomfort by providing all patients an individually adapted plan for when CP should be performed. This plan must be devised with each individual patient such that the patient can share individual needs, concerns, and questions. To make the waiting time as safe as possible, individually tailored information and contact details for the healthcare agency responsible for CP must be presented to the patients. An evidence-based nursing review has recommended better care coordination to identify the ideal timing and monitoring of late complications [16].

### Methodological discussion

Interviewing people with acquired brain damage and communication impairment entails considerable challenges. These patients often have difficulties speaking and recounting their stories, compounded by memory loss and lack of awareness of their difficulties. Loss of concentration and fatigue are common [25]. This was noticed during the interviews, which were sometimes difficult to conduct: the patients had problems sticking to the subject, talked about other matters than those asked about, had difficulties recalling events, or had difficulties expressing themselves due to speech impairment. Answers were often short and not very substantial. Because of this, researchers normally tend to exclude people with brain damage and communication disorders from their studies [25]. However, we wanted to obtain an insiders’ perspective and the views of those who had actually experienced daily life without cranial bone protection, and to gather this knowledge, having the patients tell their own stories was a possible option.

To compensate for the methodological difficulties mentioned, we included family members and care staff to contribute their understandings and views as complementary informants. As it turned out, all types of participants were mostly in agreement in their narratives within the cases. Discrepancies appeared mainly in answers concerning worries and were perhaps due to the patients´ impaired ability to remember or fully understand their situation due to brain injury. Notably, a review [23] of the reliability of having family members assess quality of life and activities of daily living (ADL) found the reliability to be high for ADL but moderate for quality of life, greater disagreement appearing with increased severity of symptoms.

The patients’ ability to communicate was not assessed before interviews other than by talking to family members when scheduling interviews. Initially, open-ended questions were asked during interviews, leading to more direct questions if patients had difficulties answering. To additionally clarify the patients’ stories, field notes were made of non-verbal communication and corrections when struggling to find the right words due to aphasia.

In studies with qualitative design, there is a risk that the researcher’s pre-understanding may influence both how questions are asked and how the analysis is performed [9]. This was a particular risk in this study when the patients answered briefly, leading to follow-up questions. Numerous direct questions lead to the danger of the result simply confirming the author’s pre-understanding [19]. By involving all authors in formulating the interview guide as well as in the analysis process, the influence of any one person’s pre-understanding was reduced. Also, the fact that one of the authors has no experience of working with this particular patient group was a strength in this respect. Whiffin et al. [29] have argued that qualitative research methods are underused in the neurosurgical field and are needed to help advance person-centered care in neurosurgery. Knowledge of the lived daily-life experiences of patients without cranial bone protection cannot be captured in any other way than through patients telling their stories, as in this study.

## Conclusion

The results show a vulnerable group of patients with brain damage and communication impairments struggling to develop new routines during a waiting period experienced as being in limbo. To make this situation safe and reduce some problems in daily life living without cranial bone protection, a person-centered approach to care, including available contact information for the correct healthcare institution and individually planned scheduling for CP, would be greatly warranted.

## Supplementary Information

Below is the link to the electronic supplementary material.

Supplementary file1 (DOCX 15 KB)

Supplementary file2 (PDF 543 KB)

## Data Availability

The data that support the findings of this study are available from the corresponding author, [HG], upon reasonable request.

## Abbreviations

DC: Decompressive craniectomy
CP: Cranioplasty
NICU: Neurointensive care unit
ICP: Intracranial pressure
GCS-M: Glasgow Coma Scale—Motor Score
GOS-E: Glasgow Outcome Scale-Extended
ADL: Activities of daily living
