# Supplementary material for: Daily life without cranial bone protection while awaiting cranioplasty: a qualitative study
Source: Acta Neurochir (Wien). 2024 Aug 9;166(1):330. doi: 10.1007/s00701-024-06217-5 (PMC11333547; doi:10.1007/s00701-024-06217-5)
Supplement: Supplementary file 1 — Supplementary file1 (DOCX 15 KB) [file 701_2024_6217_MOESM1_ESM.docx]

# Interview guide

## For all interviews

The interview will start with introductory information regarding the study and the interview setup, together with signing the consent form.

The interviews will be conducted as a reflective conversation prompted by open-ended questions, followed with probing questions to seek clarification.

- Can you give an example?
- Can you tell more about …?
- In what situations … ?

The environment around the patient can guide the opening questions, such as:

- I notice that you are wearing a helmet …
- I notice your walker …

The interview will end with summing up the conversation, giving the informant the opportunity to add content not included in the following questions.

## Questions to patients

The main question is “How is daily living affected by not having skull protection?” In cases in which participants have communication impairment due to the acquired brain damage, more direct questions will be asked, aiming to explore the following topics in relation to living without bone protection and daily living.

- How are sleep and rest affected by the absence of bone protection?
- How are activities of daily living working out?
- How are daily activities affected by the absence of bone protection?
- Is the absence of bone protection affecting social activities in any way?
- Is the absence of bone protection causing any concern or anxiety?
- How do you perceive the received information about daily living without bone protection?

Questioning will aim to ask about daily situations chronologically, starting with the morning. The questions will be about recent activities (e.g., “How did you sleep last night? Is the absence of bone protection affecting your sleep?”). Questions will be selected strategically together with family members when the patient is believed to be having difficulties managing the interview due to fatigue. The opening question will be the same for all informants in the patient group to get an understanding of their ability to communicate.

- We are going to talk about the fact that your skull bone was removed. How is that affecting your daily life?

## Questions to family members

*Questions regarding demographic data*

- The informant’s relationship to the patient
- Are the informant and the patient living together?
- Does the patient receive any homecare service, and from whom?

*Questions regarding daily life without bone protection*

The interview will be conducted using open-ended questions, with the main question being “How is your family member’s daily life affected by the absence of bone protection?” The topics to be explored will be the same as above.

## Questions to caring staff

*Questions regarding demographic data*

- The informant’s profession and previous experience of caring for patients with an absence of bone protection

*Questions regarding daily life without bone protection*

The interview will be conducted using open-ended questions, with the main question being “How is your patient’s daily life affected by the absence of bone protection?” The topics to be explored will be the same as above.
